# Measuring situation awareness in health care providers: a systematic review of measurement properties using COSMIN methodology

**DOI:** 10.1186/s13643-023-02220-6

**Published:** 2023-04-01

**Authors:** Chiman Ghaderi, Roghayeh Esmaeili, Abbas Ebadi, Mohammad Reza Amiri

**Affiliations:** 1 Department of Anesthesiology and Operating Room, Student Research Committee, School of Nursing and Midwifery, Shahid Beheshti University of Medical Sciences, Tehran, Iran; 2 Department of Operating Room, Maragheh University of Medical Sciences, Maragheh, Iran; 3 Department of Medical-Surgical, School of Nursing and Midwifery, Shahid Beheshti University of Medical Sciences, Vali Asr Ave., Niayesh Cross Road, Niayesh Complex, Tehran, Iran; 4 Behavioral Sciences Research Center, Life Style Institute, Baqiyatallah University of Medical Sciences, Tehran, Iran; 5 Nursing Faculty, Baqiyatallah University of Medical Sciences, Tehran, Iran; 6 Department of Medical Library and Information Sciences, School of Paramedicine, Hamadan University of Medical Sciences, Hamadan, Iran

**Keywords:** Situation awareness, Non-technical skills, Psychometrics, Validity, Reliability, Health care providers

## Abstract

**Background:**

Situation awareness (SA) is a key factor in accountability and ensuring patient safety in health care. SA is an essential element to research on human factors in healthcare. It is essential to identify valid instruments for measuring this concept and assessing how it is affected by interventions and educational methods.

**Methods:**

This systematic review aimed to assess the measurement properties of situation awareness instruments in health care providers’ (HCP_S_) using the COnsensus-based Standards for the selection of health Measurement INstruments (COSMIN) methodology. Four databases (Medline (through PubMed), Embase, Scopus, and Web of Science) were systematically searched. A manual search was also conducted on Google Scholar and the reference list of the included primary studies to supplement the electronic search. Studies aiming to determine the measurement properties of SA instruments or non-technical skills in HCP_S_ were included. The overall results for each measurement property were reported as sufficient, insufficient, inconsistent, or indeterminate, and the quality of evidence was reported as high, moderate, low, or very low.

**Results:**

A total of 25 studies and 15 instruments were included in the study. More than one measurement property was reported in some of the studies, and none of the studies presented all measurement properties. The most common measurement properties were content validity (12/25) and internal consistency (12/25). Cross‐cultural validity and responsiveness were not investigated in any study. Evidence quality for the measurement properties was not high in any of the 15 instruments.

**Conclusions:**

None of the instruments can be recommended as the most suitable instrument, and all instruments were classified as promising instruments in need of further psychometric assessment. This systematic review proves the dire need for the development and psychometric evaluation of instruments to measure SA in HCPs in clinical settings.

**Systematic review registration:**

PROSPERO CRD42020147349.

**Supplementary Information:**

The online version contains supplementary material available at 10.1186/s13643-023-02220-6.

## Background

In health care, situation awareness (SA) is one of the most prominent non-technical skills and a basis for appropriate clinical decision-making implicated in optimal health care providers’ (HCPs) performance, patient safety, and positive outcomes [1]. SA is an essential element to research human factors in healthcare [2]. Endsley [3] defines SA as "the perception of the elements in the environment within a volume of time and space, the comprehension of their meaning and a projection of their status in the near future".

In recent years, the number of studies on SA in nurses [4], anesthesiologists [5], surgeons [6], and students of nursing and medical majors has remarkably increased [7]. The main challenge is for HCPs with the most efficient SA education and SA assessment. Between the two, SA assessment is the more challenging [8, 9].

To improve the quality of care, decrease complications resulting from medical errors, increase patient safety, and investigate the effects of interventions on SA, researchers need to identify the most suitable way to assess SA in HCPS.

Instruments for SA measurement in health care are limited and mostly appropriated from other disciplines [10]. The previous review indicates that the Situation Awareness Global Assessment Technique (SAGAT) and the behavioral rating system-based instruments are the most common instruments for SA measurement in HCPs [4, 11], but a comprehensive appraisal of their measurement properties is not available. Research for current situation identification or outcome evaluation purposes using low quality or unknown quality measurement instruments causes a waste of resources and misdirection of further investigating and training.

This systematic review is the first study using the COSMIN methodology and an up-to-date review of instruments for SA measurement in HCPs. This study aimed to provide a comprehensive view of this instrument’s measurement properties, support evidence-based recommendations in selecting the most suitable instrument, and identify potential improvements in this field from a psychometric point of view.

This study aimed to critically evaluate and summarize the quality of measurement properties in instruments used for measuring SA in HCPs using the COSMIN methodology. Moreover, we aimed to classify the instruments into three groups of 1- instruments that are the most suitable, 2- instruments that need further studies and development, and 3- instruments that are unsuitable, and to provide explanations and reasons for this classification.

## Methods

**Fig. 1:**
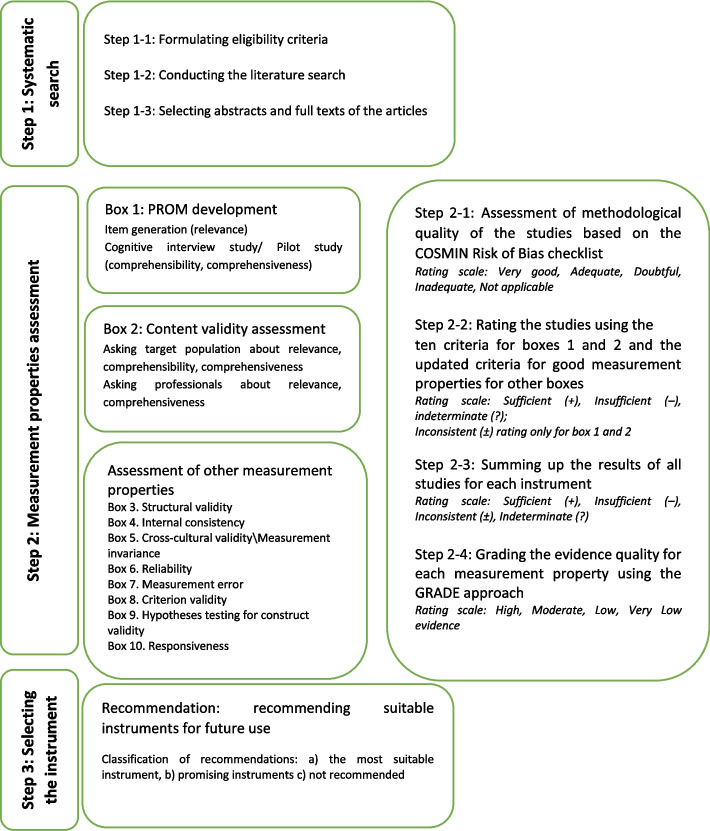
Study design according to COSMIN methodology for systematic reviews of instruments

In this systematic review, the COSMIN methodology for systematic reviews of Patient‐Reported Outcome Measures (PROM_S_) and the Preferred Reporting Items for Systematic Reviews and Meta-Analyses (PRISMA) were used as methodological guides [12–15]. The protocol of this systematic review has been registered in PROSPERO (CRD42020147349). The PRISMA checklist completed for the present study is presented in the (Additional file 1). This study was conducted in three steps (Fig. 1), explained below.

### Step 1: systematic literature search

#### Step 1–1: formulating eligibility criteria

The inclusion criteria were as follows: 1- articles aiming to develop and determine measurement properties of instruments for measurement of SA or measuring non-technical skills (assessing SA as one of the dimensions of them: according to the guidelines of COSMIN methodology for systematic reviews of Patient‐Reported Outcome Measures; subscales of instruments can also be assessed using the COSMIN Risk of Bias checklist) in HCPs in clinical or simulated settings, and 2- articles published and instruments developed in English.

Studies on other non-technical skills or in populations different from that of the present study, as well as studies whose full texts were not accessible or were in the form of unpublished manuscripts, conference proceedings, and dissertations, were excluded.

#### Step 1–2: conducting the literature search

Four databases (Medline (through PubMed), Embase, Scopus, and Web of Science) were systematically searched from inception to December 2020 for peer-reviewed articles. A manual search on Google Scholar and the reference list of the included primary studies were also conducted to supplement the electronic search. The search strategy was developed with the assistance of a health sciences librarian and conducted using a combination of keywords and database-specific subject headings related to SA and psychometric properties (Additional file 2).

#### Step 1–3: selecting abstracts and full texts of the articles

The duplicates were omitted after transferring the search results to EndNote X7 (Thomson Reuters, Philadelphia, PA). The results were analyzed by two researchers independently based on titles and abstracts to identify the eligible articles. Then, the full text of the articles whose abstracts were screened in the previous step were investigated to determine if they met the inclusion criteria.

All the above steps were conducted independently by two researchers to reduce bias, and in the case of different opinions on an article, disagreements were resolved through discussion and consensus.

### Step 2: assessment of measurement properties

Assessment of the instruments' measurement properties was done in 4 steps: 1) Assessment of the methodological quality of the studies based on the COSMIN Risk of Bias checklist, 2) rating results for single studies using the updated criteria for good measurement properties, 3) summing up the results of all studies for each instrument, and 4) grading the evidence quality for each measurement property using the GRADE approach.

In this step, all assessments were made by two reviewers independently, and disagreements were resolved through discussion or consulting a third person.

#### Step 2–1: assessment of the methodological quality of studies

The COSMIN Risk of Bias checklist was used for assessing the methodological quality of the study measurement properties. The COSMIN Risk of Bias Checklist has 10 boxes (see Fig. 1) for assessment. To assess the methodological quality of each study, first, the measurement properties were specified, and then, relevant boxes were selected. Each measurement standard was scored using a 4-point scale consisting of "very good," "adequate," "doubtful," and "inadequate"; moreover, the overall score of each box was determined by the lowest score of each item based on "the worst score counts" principle.

#### Step 2–2: rating the results of single studies

Rating of the results for single studies for the instrument development and content validity boxes was done separately based on 10 criteria (5 criteria for relevance, 1 for comprehensiveness, and 4 for comprehensibility). This method is thoroughly explained in the COSMIN Methodology for Assessing the Content Validity of PROMs. Rating of the studies for other measurement properties was done separately using the updated criteria for good measurement properties, and the results were rated sufficient (+), insufficient (-), or indeterminate (?) (Additional file 3).

#### Step 2–3: summing up the results of all studies for each instrument

All of the results of the studies were qualitatively summed up regarding each measurement property for each instrument, and using the 75% agreement rule; the results were rated sufficient (+), insufficient (-), inconsistent (±), or indeterminate (?). In this step, the focus is on the instrument, while the previous step focuses on the results of the single studies.

In this step, all the results regarding instrument development, content validity, and reviewers’ rating were qualitatively summed up for the overall rating of the relevance, comprehensiveness, and comprehensibility of the instrument. The results were rated sufficient (+), insufficient (-), or inconsistent (±). Since the reviewers' qualitative rating, as + , -, or ± , was possible in this step, the indeterminate (?) rating can be ignored for content validity.

#### Step 2–4: grading the evidence quality for each measurement property

In the final step, the summarized evidence was graded using Grading of Recommendations Assessment, Development and Evaluation (GRADE). This was done to determine the overall quality of the instrument, and evidence quality is graded as high, moderate, low, or very low.

This method is thoroughly explained in the COSMIN methodology for systematic reviews. In summary, 4 factors are considered in this rating: a) risk of bias (limitations in methodological quality of studies), b) inconsistency (unjustifiable heterogeneity in the results of studies), c) indirectness (evidence from populations different from the target population in the review), and d) imprecision (small number of samples). (Additional file 4).

### Step 3: selecting the instrument

Selecting suitable instruments was done based on a combination of the results of steps 2–3 and step 2–4 in the assessment of content validity and other measurement properties. The recommendations were categorized into three groups: a) the most suitable instrument (high-quality evidence for sufficient content validity in terms of relevance, grading the evidence quality for each measurement property, and at least low-quality evidence of a sufficient internal consistency), b) promising instruments needing more psychometric studies (instruments not classified in a and c), c) not recommended (instruments with high-quality evidence of insufficient psychometric properties).

This study did not assess interpretability and feasibility since they are not considered measurement properties.

## Results

### The systematic literature search

**Fig. 2:**
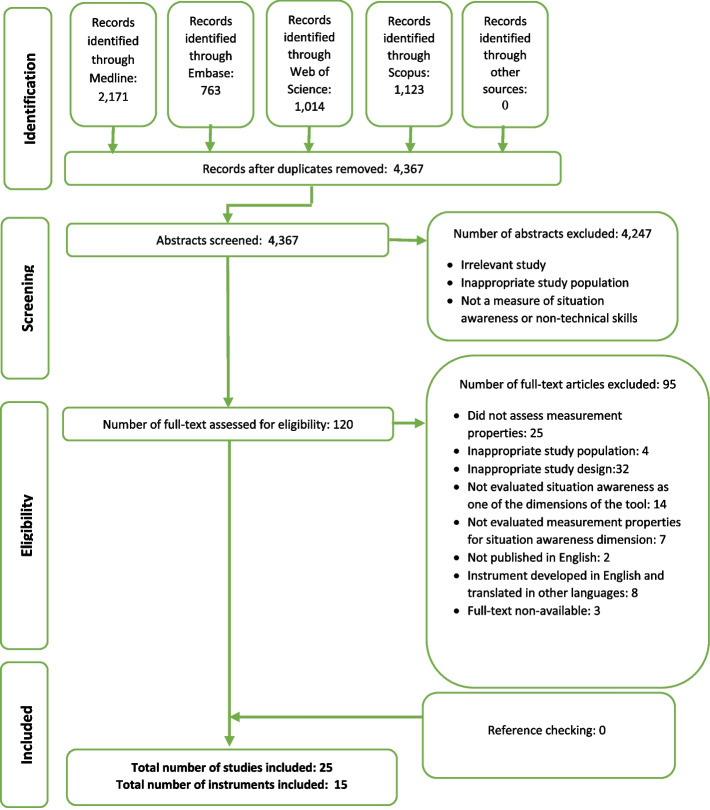
Flow diagram of the reviewing process according to PRISMA

After removing duplicates, a total of 4367 abstracts were recovered from 4 databases. After checking the titles and abstracts, 4247 articles were excluded due to irrelevance and ineligibility. After the assessment of 120 full-text artic eligible articles, 25 eligible articles, and 15 eligible instruments were included in the study. The flow diagram of identifying and assessing the articles is presented in Fig. 2.

The inter-rater reliability was acceptable based on Altman [16]. The weighted kappa coefficient for the primary screening was 0.76 (95% confidence interval [CI] = 0.62–0.95) and 0.77 for the secondary screening (95% CI = 0.67–0.85).

Four instruments had assessed SA, and 11 had assessed non-technical skills, of which SA was a dimension of the instrument in different HCP_S_. Moreover, 9 studies were conducted in clinical and 14 in simulated settings. One study was conducted in both clinical and simulated settings, and in one study research setting was not reported. More than half of the studies (16 studies) had assessed SA and non-technical skills in HCPs in operating rooms, and others had been conducted in clinical settings or in simulated trauma, acute and intensive care units. Fourteen instruments were observational checklists, and only SAGAT was the pen-and-paper version of the instrument; regarding one of the instruments, it was not determined if it was observational or self-report. A summary of the information on the studies and instruments is presented in Table 1.

**Table 1:** Characteristics of the instruments used to measure SA in HCPs and description of the included studies

**Instrument name**	**Type of measure**	**Number of subscales**	**Total items**	**Response Options**	**Reference** **country**	**Study participants**	**Setting (clinical vs. simulation)**	**Measurement properties**
Situation Awareness Global Assessment Technique (SAGAT)	NR	3	39	NR	Dishman et al., 2020 [9] USA	49 Nurse anesthetists 7 experts	Simulation (scenario of induction of general anesthesia)	Content validity
	Open-ended questions		3	Correct 1, Incorrect 0, Partial correct 0.5	Gardner et al., 2017 [17] USA	43 medical students	Simulation (advanced Cardiac life support scenarios)	Criterion validity
	Pen-and-paper version of the instrument		31	Yes/No for level 1; 2 possible answers for level 3	Lavoie et al., 2016 [18] Canada	234 nursing students 15 critical care experts	Simulation (patient deterioration simulation scenario)	Content validity Internal consistency
	Pen-and-paper version of the instrument		7	Answers were based on factual aspects and expert opinion	Hogan et al., 2006 [19] Canada	16 surgeons and residents	Simulation (human patient Simulator and trauma scenarios)	Content validity Internal consistency Convergent validity
Team resuscitation situation awareness tool	Observational checklist	7	7	5-point Likert scale:	O'Neill et al., 2018 [20] Canada	42 teams and 242 HCP_S_ (physicians and nurses) 13 experts	Simulation (simulated pediatric resuscitation events)	Content validity Inter-rater reliability Criterion validit
Team Situation Awareness Global Assessment Technique (TSAGAT)	Observational checklist	3	50	3-point Likert scale	Crozier et al., 2015 [21] Canada	12 HCP_S_ (physicians, nurses and students) 2 independent raters	Simulation (trauma resuscitation scenarios using HPS)	Convergent validity Known-groups validity Inter-rater reliability
Situation awareness (SA) assessment tool	Observational checklis	3	14	NR	Frere et al., 2017 [8] Ireland	2 expert raters	Simulation (OSCE in 9 medical specialties)	Internal consistency Inter-rater reliability
Non-Technical Skills for Surgeons tool (NOTSS)	Observational checklist	4	12	4-point rating Scale	Jung et al., 2020 [22] Canada	5 experts	Clinical (observing recordings of actual OR)	Known-groups validity Inter-rater reliability
					Yule et al., 2018 [23] UK-USA	255 surgeons in 2 groups	Simulation (video-based simulated crisis scenario)	Structural validity Internal consistency Criterion validity
					Crossley et al., 2011 [24] UK	85 surgeons 100 assessor	Clinical (OR)	Content validity Structural validity Internal consistency
Non-Technical Skills for Surgeons (NOTSS) tool		5	14		Yule et al., 2008 [25] UK	44 surgeons	Simulation (video-based simulated scenario)	Internal consistency Inter-rater reliability
					Yule et al., 2006 [26] UK		Clinical (OR)	Development Study
Non-Technical Skills for Urological Surgeons (NoTSUS)	Observational checklist	5	13	5-point Likert scale	Aydın et al., 2020 [27] UK	43 trainees and 19 specialists 5 expert raters	Simulation (the full immersion simulation ‘Igloo’ environment)	Criterion validity Inter-rater reliability
Anesthetists' Non-Technical Skills System (ANTS)	Observational checklist	4	15	4-point rating scale	Fletcher et al., 2003 [28] UK	50 anesthetists	Simulation (simulated anesthetic scenarios)	Content validity Internal consistency Inter-rater reliability
Anesthetists' Non-Technical Skills System (ANTS)	Observational checklist	4	15	4-point rating scale	Graham et al., 2010 [29] Australia	26 anesthetists	Clinical (videos of real-time and routine anesthesia)	Internal consistency Inter-rater reliability
Anaesthetic Non-technical Skills for Anesthetic Practitioners System (ANTS-AP)	Observational checklist	3	9	4-point rating scale	Rutherford et al.,2015 [30] UK	48 anesthetic practitioners	Simulation (Simulated anesthetic scenarios in OR	Content validity Internal consistency Reliability Inter-rater reliability
Trauma Non-Technical Skills (T-NOTECHS) tool	Observational checklist	5	5	5-point scale	van Maarseveen et al., 2020 [31] Netherland	18 recorded videos of resuscitations team 3 assessors	Clinical (trauma center)	Reliability Inter-rater reliability
					Steinemann et al.,2012 [32] USA	44 observations for simulated and 48 for actual resuscitations by 2–3 raters	Both clinical and simulation setting	Development Study Inter-rater reliability
Oxford Non-Technical Skills scale (NOTECHS)	Observational checklist	4	16	4-point rating scale	Mishra et al., 2009 [33] UK	65 OR teams 2–3 expert raters	Clinical (OR)	Content validity Reliability convergent validity Inter-rater reliability
Oxford Non-Technical Skills scale (NOTECHS II)				8-point rating scale	Robertson et al.,2014 [34] UK	297 OR members	Clinical (OR)	Content validity Known-groups validity Inter-rater reliability
Interpersonal and Cognitive Assessment for Robotic Surgery rating system (ICARS)	Observational checklist	4	28	5-point rating scale	Raison et al., 2017 [35] UK	16 expert surgeons 73 surgeons	Simulation (ureterovesical anastomosis within a simulated OR)	Content validity Internal consistency Inter-rater reliability
Explicit professional oral communication tool (EPOC)	Observational checklist	6	35	NR	Kemper et al., 2013 [36] Netherland	378 ED members 1144 ICU members 2 independent observers	Clinical (ED and ICU)	Measurement error Inter-rater reliability
Scrub Practitioners’ List of Intraoperative Non-Technical Skills (SPLINTS)	Observational checklist	3	9	4-point Likert scale	Loh et al., 2019 [37] Singapore	30 scrub nurses 10 expert raters	Clinical (OR)	Content validity Internal consistency Reliability Convergent validity Inter-rater reliability
				Binary scale	Mitchell et al., 2012 [38] UK	25 scrub nurses 9 surgeons	NR	Development Study
Ottawa Global Rating Scale (GRS)	Observational checklist	8	8	7-point rating scale	Kim et al., 2006 [39] Canada	59 medical residents 3 raters	Simulation (ICU; ED; PACU)	Content validity Internal consistency Inter-rater reliability

*NR* Not Reported, *OR* Operating Room, *NTS* Non-Technical Skills, *ED* Emergency Department, *ICU* Intensive Care Unit, *OSCE* objective structured clinical examination, *N/A* Not Applicable (skill not required for the given clinical sitting), *PACU* post anesthesia care unit

### Measurement properties assessment

The methodologic quality of the 25 included studies (Step 2–1) was assessed using the COSMIN Risk of Bias checklist. In some studies, more than one psychometric property was measured. Also, more than one psychometric study was conducted on four instruments in various research settings and conditions. The studies' most frequently measured psychometric properties were content validity (12 studies) and internal consistency (12 studies). A small number of the studies had investigated hypothesis testing for construct validity (6 studies), criterion validity and reliability (4 studies), and structural validity (2 studies), and only one study had assessed measurement error. Cross‐cultural validity and responsiveness were not investigated in any study.

The results of the methodological quality assessment (step 2–1) and the rating of the results of the single studies for each measurement property (step 2–2) are presented in Additional files 5 and 6, respectively.

Table 2 presents the results of all the studies for each instrument (2–3) and the grading of the evidence quality for each measurement property (steps 2–4). According to evidence on the content validity of the 15 instruments, only SAGAT (Dishman, 2020) had high-quality evidence of the sufficiency of its relevance; however, the evidence quality in this study was low and very low for the sufficiency of its comprehensiveness and comprehensibility, respectively. Other than this instrument, the evidence quality was not high for the sufficiency or insufficiency of measurement properties of any of the 15 instruments. In cases where the sum of the results for one measurement property was indeterminate (?), the evidence quality was reported as not evaluable (NE) due to insufficient evidence. In some studies, due to the lack of assessment or report of measurement properties, the sum of results and evidence quality was reported as NR (not reported).

**Table 2:** Quality of evidence (and Overall Rating) for measurement properties of the instruments

**Instrument**	**Content validity**			**Structural validity**	**Internal consistency**	**Reliability**	**Measurement error**	**Criterion validity**	**Hypothesis Testing**
	**Relevance**	**Comprehensiveness**	**Comprehensibility**						
**SAGAT (Dishman 2020)**	High (+)	Low (+)	Very Low (+)	NR	NR	NR	NR	NR	NR
**SAGAT (Gardner 2017)**	Low (+)	Very Low (-)	Very Low (-)	NR	NR	NR	NR	Very Low (+)	NR
**SAGAT (Lavoie 2016)**	Moderate (+)	Low (+)	Low (+)	NR	Very Low (-)	NR	NR	NR	NR
**SAGAT (Hogan 2006)**	Very Low (-)	Very Low (+)	Very Low (-)	NR	Very Low (?)	NR	NR	NR	Very Low (?)
^**a**^**Unnamed (O'Neill 2018)**	Moderate (+)	Low (+)	Low (+)	NR	NR	NR	NR	NE (?)	NR
**TSAGAT (Crozier 2015)**	Moderate (-)	Low (+)	Low (+)	NR	NR	NR	NR	NR	Very Low (+)
^**b**^**Unnamed (Frere 2017)**	Low (-)	Very Low (-)	Very Low (-)	NR	Moderate (+)	NR	NR	NR	NR
**NOTSS (Jung 2020; Yule 2018; Crossley 2011; Yule 2008; Yule 2006)**	Moderate (+)	Moderate (+)	Moderate (+)	Moderate (±)	Moderate (±)	NR	NR	NE (?)	Moderate (+)
**NoTSUS (Aydın 2020)**	Moderate (-)	Very Low (-)	Very Low (-)	NR	NR	NR	NR	Moderate (+)	NR
**ANTS (Fletcher 2003; Graham 2010)**	Moderate (+)	Moderate (+)	Moderate (+)	NR	Low (+)	NR	NR	NR	NR
**ANTS-AP (Rutherford 2015)**	Moderate (+)	Moderate (+)	Moderate (+)	NR	Low (+)	Low (-)	NR	NR	NR
**T-NOTECHS (van Maarseveen 2020; Steinemann 2012)**	Moderate (+)	Low (-)	Very Low (+)	NR	NR	Very Low (+)	NR	NR	NR
**NOTECHS (Mishra 2009)**	Moderate (+)	Low (+)	Low (+)	NR	NR	NE (?)	NR	NR	Very Low (+)
**NOTECHS II (Robertson 2014)**	Moderate (+)	Low (+)	Low (+)	NR	NR	NR	NR	NR	Moderate (+)
**ICARS (Raison 2017)**	Moderate (+)	Low (+)	Very Low (+)	NR	Moderate (+)	NR	NR	NR	Moderate (+)
**EPOC (Kemper 2013)**	Moderate (+)	Low (-)	Very Low (-)	NR	NR	NR	NE (?)	NR	NR
**SPLINTS (Loh 2019; Mitchell 2011)**	Moderate (+)	Low (+)	Low (-)	NR	Low (+)	Very Low (+)	NR	NR	Very Low (+)
**Ottawa GRS (Kim 2006)**	Low (-)	Low (-)	Very Low (-)	NR	NE (?)	NR	NR	NR	NR

See Table 1 for the full name of instruments

Rating scale for overall rating: Sufficient (+), Insufficient (–), Inconsistent (±), Indeterminate (?)

Rating scale for grading the quality of evidence: High, Moderate, Low, Very low (see Additional file 4)

Cross-cultural validity and Responsiveness, were not assessed for any of the instruments, were deleted

*NR* not reported, *NE* not evaluated

^a^Team resuscitation situation awareness tool

^b^Situation awareness (SA) assessment tool

The inter-rater reliability in quality assessment was acceptable: The kappa coefficient was 0.67 (95% CI = 0.55–0.8) for step 2–1, 0.62 (95% CI = 0.42–0.75) for step 2–2, 0.74 (95% CI = 0.63–0.83) for step 2–3, and 0.74 (95% CI = 0.65–0.87) for step 2–4.

### Quality of psychometric properties

Four included instruments were assessed in numerous studies, as identified in this systematic review, and other instruments were in only one. The measurement properties of these instruments were summarized and evaluated on the basis of criteria for good measurement properties, and the quality of evidence was graded using a modified GRADE approach. The results of the evidence synthesis are presented in Table 3.

**Table 3:** Summary of findings

	**Summarised results**	**Overall rating**	**Quality of evidence**
**SAGAT (Dishman 2020)** Content validity	Relevance + Comprehensiveness + Comprehensibility +	Sufficient	Moderate ^c^
**SAGAT (Gardner 2017)** Content validity Criterion validity	Relevance + Comprehensiveness – Comprehensibility – SA significantly predicted teamwork ratings (first scenario R2 = 0.50; second scenario R2 = 0.55)	Inconsistent Sufficient	Very Low ^a, c^ Very Low ^a, c^
**SAGAT (Lavoie 2016)** Content validity Internal consistency	Relevance + Comprehensiveness + Comprehensibility + Cronbach’s alpha rating for total scale: 0.64	Sufficient Insufficient	Moderate ^a^ Very Low ^a^
**SAGAT (Hogan 2006)** Content validity Internal consistency Hypothesis Testing	Relevance – Comprehensiveness + Comprehensibility – Cronbach’s alpha rating for total scale: 0.76 Hypothesis confirmed	Inconsistent Indeterminate Indeterminate	Very Low ^a, c^ Very Low ^a, c^ Very Low ^a, c^
**Unnamed (O'Neill 2018)** Content validity Criterion validity	Relevance + Comprehensiveness + Comprehensibility + Area under the curve and Correlation not reported	Sufficient Indeterminate	Low ^a^ Not evaluated
**TSAGAT** Content validity Hypothesis Testing	Relevance – Comprehensiveness + Comprehensibility + Hypothesis confirmed	Inconsistent Sufficient	Low ^a^ Very Low ^a^
**Unnamed (Frere 2017)** Content validity Internal consistency	Relevance – Comprehensiveness – Comprehensibility – Cronbach’s alpha rating ≥ 0.7	Insufficient Sufficient	Low ^a, c^ Moderate ^c^
**NOTSS** Content validity Structural validity Internal consistency Criterion validity Hypothesis Testing	Relevance + Comprehensiveness + Comprehensibility + Confirmatory factor analysis demonstrated an acceptable model fit RMSEA: 0.094–0.213; Comparative fit index: 0.554–0.944; χ2/d.f: 1.69–4.55; Multidimensional scale (4 subscales) Cronbach’s alpha rating for total scale from 0.7 to 0.95 Area under the curve and Correlation not reported 3 out of 4 Hypotheses confirmed	Sufficient Inconsistent Inconsistent Indeterminate Sufficient	Moderate ^c^ Moderate ^b^ Moderate ^b^ Not evaluated Moderate ^b^
**NoTSUS** Content validity Criterion validity	Relevance – Comprehensiveness – Comprehensibility – Correlation of the NoTSS and NoTSUS scores: 0.88–0.93	Insufficient Sufficient	Low ^a, c^ Moderate ^c^
**ANTS** Content validity Internal consistency	Relevance + Comprehensiveness + Comprehensibility + Cronbach’s alpha for situation awareness subscale: 0.87 Cronbach’s alpha rating for total scale from 0.79 to 0.86	Sufficient Sufficient	Moderate ^c^ Low ^a, c^
**ANTS-AP** Content validity Internal consistency Reliability	Relevance + Comprehensiveness + Comprehensibility + Cronbach’s alpha for situation awareness subscale: 0.78 Interclass Correlation Coefficient for situation awareness subscale: 0.54	Sufficient Sufficient Insufficient	Moderate ^c^ Low ^a, c^ Low ^a, c^
**T-NOTECHS** Content validity Reliability	Relevance + Comprehensiveness – Comprehensibility + Interclass Correlation Coefficient for situation awareness subscale: 0.87 Interclass Correlation Coefficient for total scale: 0.71	Inconsistent Sufficient	Very Low ^a, c^ Very Low ^a, c^
**NOTECHS** Content validity Reliability	Relevance + Comprehensiveness + Comprehensibility + Interclass Correlation Coefficient not reported	Sufficient Indeterminate	Very Low ^a, c^ Not evaluated
**NOTECHS II** Content validity Hypothesis Testing	Relevance + Comprehensiveness + Comprehensibility + Hypothesis confirmed	Sufficient Sufficient	Very Low ^a, c^ Very Low ^a, c^
**ICARS** Content validity Internal consistency Hypothesis Testing	Relevance + Comprehensiveness + Comprehensibility + Cronbach’s alpha rating for total scale: 0.92 Hypothesis confirmed	Sufficient Sufficient Sufficient	Very Low ^a, c^ Moderate ^c^ Moderate ^c^
**EPOC** Content validity Measurement error Hypothesis Testing	Relevance + Comprehensiveness – Comprehensibility – Measurement error not reported Hypothesis confirmed	Inconsistent Indeterminate Sufficient	Very Low ^a^ Not evaluated Moderate ^a^
**SPLINTS** Content validity Internal consistency Reliability Hypothesis Testing	Relevance + Comprehensiveness + Comprehensibility – Cronbach’s alpha for situation awareness subscale: 0.7 Interclass Correlation Coefficient for total scale: 0.85 Hypothesis confirmed	Inconsistent Sufficient Sufficient Sufficient	Very Low ^a^ Low ^c^ Very Low ^a, c^ Very Low ^a, c^
**Ottawa GRS** Content validity Internal consistency	Relevance – Comprehensiveness – Comprehensibility – Cronbach’s alpha not reported	Insufficient Indeterminate	Very Low ^a, c^ Not evaluated

See Table 1 for the full name of instruments

Rating scale for overall rating: Sufficient (+), Insufficient (–), Inconsistent (±), Indeterminate (?)

“a” downgrading for Risk of Bias; “b” downgrading for inconsistency; “c” downgrading for imprecision; “d” downgrading for indirectness

#### Non-technical skills for surgeons tool

Moderate quality of evidence (due to imprecision) was found for sufficient content validity. The NOTSS scale results showed moderate quality of evidence for inconsistent structural validity. Cronbach’s alpha for each of the subscales was not reported in the five studies in which internal consistency was evaluated, resulting in moderate quality of evidence for inconsistent internal consistency. Three of the four hypotheses were confirmed, resulting in moderate-quality of evidence for sufficient construct validity. The quality of evidence for the indeterminate criterion validity was not graded, because the results of the study on criterion validity were missing.

#### Anesthetists' non-technical skills system

Moderate quality of evidence was found for sufficient content validity. Low evidence for sufficient internal consistency was shown in ANTS. Cronbach’s alpha for the situation awareness subscale was reported in one of two studies.

#### Trauma non-technical skills tool

Very low-quality evidence for inconsistent content validity was found. T-NOTECHS showed a very low quality of evidence for sufficient reliability, due to the low sample size in the study and methodological flaws.

#### Scrub practitioners’ list of intraoperative non-technical skills

Very low-quality evidence for inconsistent content validity was found. Very low-quality evidence was found for sufficient reliability and sufficient construct validity. Low evidence for sufficient internal consistency was shown in SPLINTS. Cronbach’s alpha for the situation awareness subscale was reported in one of two studies.

Also, the evidence of measurement properties for SAGAT as tools for direct assessment of SA is highlighted below.

#### Situation awareness global assessment technique

Moderate quality of evidence for sufficient content validity was found in the studies by Dishman 2020 and Lavoie 2016. Very low-quality evidence for inconsistent content validity was found in the studies by Gardner 2017 and Hogan 2006. Very low quality of evidence was determined for sufficient criterion validity, insufficient and indeterminate internal consistency, and indeterminate hypothesis testing.

Table 3 provides an overview of the findings and all instruments above and others that were tested in only one study, as identified in this systematic review.

### Instrument selection

Due to a lack of high-quality evidence of sufficient content validity in all three aspects, none of the instruments are recommended as the most suitable; however, no high-quality evidence existed proving insufficient psychometric properties. All instruments were classified as promising and more psychometric studies should be conducted on them.

## Discussion

This systematic review was a comprehensive measurement property review of instruments for SA measurement in HCPs, and it provides evidence-based recommendations for selecting suitable, reliable, and valid instruments. Twenty-five studies were identified for assessing the measurement properties of 15 instruments based on study objectives.

Five psychometric studies were conducted on SAGAT; SAGAT provides the possibility of collecting data on the three SA levels through observation or direct questions in simulation, and the content of the questions is different based on the situation [40]. Due to the difference in the content of the questions, these 5 studies were evaluated separately, and we could not sum up the results of all the studies for each instrument (step 2–3) for SAGAT. TSAGAT and team resuscitation situation awareness tool measured team SA [20, 21], but they were included since each person's SA was measured separately, and team SA was reported as the sum of individual scores in these studies.

In 18 studies, psychometric properties of instruments based on the behavioral rating system were assessed, and SA was introduced as a dimension of these instruments. In the assessment of measurement property quality, the SA subscale was assessed, and evidence quality for this instrument is only applicable to the SA subscale; the overall rating of the instrument for all subscales might be different from the present study results. These instruments were mainly based on the behavioral method, and one potential concern for behavioral rating systems is that subjective rating is susceptible to error; however, the objectivity of the instrument and inter-rater reliability can be improved through education [8, 20].

Psychometric assessment of the instruments in more than half of the studies was done in simulated environments (14 studies), and 14 instruments were observational checklists. The results of direct measurement of performance in a simulated environment and quantifying them to measure SA levels are essential but not sufficient and may not apply to clinical settings due to the existing complications [4].

Most of the studies were conducted in anesthesiology and operating room settings. Anesthesiology is a dynamic medical specialty with rapid and significant changes; moreover, operating rooms are complex settings with many specialties and different fields and instruments, necessitating a high level of SA. In fact, all medical specialties and HCPs need SA [5, 41, 42]. None of the studies had assessed all the measurement properties highlighted in the COSMIN methodology; also, there was only one psychometric study available for many instruments; therefore, the results on the instrument are indeterminate without a thorough evaluation of psychometric properties, validity, and reliability.

Some studies investigated content validity. In the present review, only Dishman (2020) had assessed content validity from both viewpoints, while the other studies had assessed it only from experts' and professionals' viewpoints. Content validity is the most prominent measurement property that must be considered and, when the contents of an instrument are good representatives of a construct, that instrument is more likely to achieve its assessment goals [43, 44].

Structural validity was only assessed for NOTSS in two studies [23, 24]. In the methodological quality assessment of structural validity, both studies were rated as very good since the COSMIN risk of bias checklist states that exploratory factor analysis (EFA) or confirmatory factor analysis (CFA) should be used; however, since no psychometric data were provided in the next steps, the overall rating of this validity for NOTSS, with moderate evidence quality, was reported as inconsistent. Structural validity is best assessed using the CFA method. Structural validity concerns which dimensions of the construct are assessed using the instrument and if these dimensions are in line with the theory [43, 45]. SA was defined according to Endsley's theory in the included studies, but structural validity was not reported using CFA in these two studies, or no correct reports of this method were reported.

The most frequently assessed measurement property in the studies regarding reliability was internal consistency, and most authors had used it as the only reliability index, which is not enough [43]. Internal consistency is not suitable for assessing the internal consistency of formative measures [46, 47]. Fourteen instruments were checklists, classified as formative measures, and their inter-rater reliability is a more suitable method for assessing their reliability [43, 47]. However, this reliability assessment method cannot be evaluated using the COSMIN Risk of Bias Checklist. Due to its importance in the reliability assessment of formative instruments, it can be considered in developing the COSMIN Risk of Bias Checklist.

Four studies had reported a psychometric evaluation of criterion validity; however, due to the lack of a gold standard, evidence quality was very low or not evaluable for 3 of these studies. Evidence quality for being sufficient was moderate only for NoTSUS, whose gold standard was NOTSS [48]. A gold standard instrument of a similar construct and comparing its scores with the instrument being evaluated is necessary to investigate criterion validity [49].

Hypothesis testing for the instruments was done in 6 studies; the evidence quality for the sufficiency of the overall rating of this measurement property was not high in these studies, and evidence quality was moderate for hypothesis testing of NOTECHS II and ICARS. There are various methods for hypothesis testing, but only convergent validity and known-groups validity can be assessed for hypothesis testing in the COSMIN risk of bias checklist, and the methodological quality of other methods of hypothesis testing is not evaluable [43, 49]. However, known-group validity is the best method for formative instruments [46], and 3/6 of studies had used this method for hypothesis testing.

Some of the COSMIN measurement properties might not apply to all studies; for instance, cross-cultural validity was not assessed in any of the included studies since all the instruments were developed in English and not translated from other languages, and this index did not need evaluation [13, 14].

Properties such as measurement error were assessed in only one study [36]. Understanding indices such as minimal important change (MIC) or smallest detectable change (SDC) is important in measuring scores. With this information, we can realize if a change in scores in people's performance represents valid and real change and if the change is insignificant or significant. In the present review, none of the studies reported the values of SDC and MIC. Responsiveness of an instrument refers to its ability to detect a change over time in the construct being measured, and none of the studies had assessed this issue in the present review [43, 50].

In health care, SA is an abstract concept requiring valid and reliable instruments to ensure research quality. The results of this review do not indicate that the present instruments are inefficient, but suggest that investigations of high methodological quality are required to suitably assess their measurement properties.

SAGAT is an objective instrument that develops based on Goal-Directed Task Analysis. One disadvantage of SAGAT is that it cannot easily be used in other conditions. The development of context-general measures of SA can help data collection and generalizability and more measurement properties research can be accomplished on them.

### Strengths and limitations

To our knowledge, no systematic review has provided a thorough and precise assessment of the methodological quality of existing studies on SA in HCPs or their results based on the measurement properties recommended in the COSMIN guideline. There is still a lack of information on the measurement properties of the instruments assessed in this study, and none of the instruments were recommended as the most suitable. This systematic review highlights the dire need for precise SA measurement instruments.

The gaps highlighted in this systematic review regarding measurement property assessment can be used in designing new studies on the development or psychometric assessment of this instrument. Moreover, qualitative studies are needed to assess content validity in three aspects of relevance, comprehensiveness, and comprehensibility of an instrument from the viewpoint of the target population and experts. Interventional studies can assess the responsiveness and predictive validity of measures. Authors of measurement studies should provide more precise reports on the methods used to assess validity and reliability, hypothesis testing, measurement error, and relevant details [13–15].

One of the limitations of our study was reviewing only English articles and excluding gray literature. Although after the systematic search of the databases, the reference lists of the included articles were also manually searched, some of the studies may have been missed due to publication bias. Also, since 10 properties were assessed according to the COSMIN methodology, other measurement properties might have been missed due to selective reporting bias.

## Conclusion

This systematic review assessed the measurement properties of 15 SA measurement instruments using the COSMIN methodology. According to the results, evidence on these instruments is limited, and most of them have insufficient evidence quality. It seems that research on measures of SA in health care is growing.

Endsley's model is the most commonly used in health care. More importantly, it emphasizes the possibility of abstraction at all three levels. To further identify and clarify the concept of SA in the HCP_S_ as the basic step for instrument development, it is necessary that more studies on the concept of SA be conducted on HCP_S_.

We hope that major shortcomings will be addressed using this systematic review. More studies are needed to develop new instruments specific to SA and not SA as a subscale of non-technical skills. Further assessment of measurement properties of the current instruments based on the COSMIN methodology and precise reports on measurement properties and methods used in the studies is necessary.

## Supplementary Information

**Additional file 1.** PRISMA checklist.

**Additional file 2.** Search Strategy.

**Additional file 3.** Guidance of rating the results of single studies based on COSMIN methodology.

**Additional file 4.** Guidance of grading the evidence quality for each measurement property based on COSMIN methodology.

**Additional file 5.** Methodological quality assessment 0f studies on psychometric properties of the included instruments.

**Additional file 6.** Quality of content validity (per PROM development and Content validity study, and Rating of reviewers) and other psychometric properties per study.

## Data Availability

Not applicable.

## Abbreviations

COSMIN: COnsensus-based Standards for the selection of health Measurement Instruments
SA: Situation awareness
HCPs: Health care providers
SAGAT: Situation Awareness Global Assessment Technique

## References

[1] Despins LA (2018). Advancing Situation Awareness Research. West J Nurs Res.

[2] Prineas S, Mosier K, Mirko C, Guicciardi S, Donaldson L, Ricciardi W, Sheridan S, Tartaglia R (2021). Non-technical Skills in Healthcare. Textbook of Patient Safety and Clinical Risk Management.

[3] Endsley MR (1995). Measurement of situation awareness in dynamic systems. Hum Factors.

[4] Orique SB, Despins L (2018). Evaluating Situation Awareness: An Integrative Review. West J Nurs Res.

[5] Schulz CM, Endsley MR, Kochs EF, Gelb AW, Wagner KJ (2013). Situation Awareness in Anesthesia: Concept and Research. Anesthesiology.

[6] O’Dea A, Morris M, O’Keeffe D. Experiential Training for Situation Awareness in the Operating Room. JAMA Surg. 2022;157(1):66-7. 10.1001/jamasurg.2021.4886 34757381

[7] Walshe N, Crowley CM, O'Brien S, Browne JP, Hegarty JM (2019). Educational interventions to enhance situation awareness: a systematic review and meta-analysis. Simulation in Healthcare.

[8] Frere M, Tepper J, Fischer M, Kennedy K, Kropmans T. Measuring situation awareness in medical education objective structured clinical examination guides. Educ Health Change Learn Pract. 2017;30(3):193–7. 10.4103/efh.EfH_306_16. 10.4103/efh.EfH_306_16 29786019

[9] Dishman D, Fallacaro MD, Damico N, Wright MC. Adaptation and Validation of the Situation Awareness Global Assessment Technique for Nurse Anesthesia Graduate Students. Clin Simul Nurs. 2020;43:35–43. 10.1016/j.ecns.2020.02.003.

[10] Sitterding MC, Broome ME, Everett LQ, Ebright P (2012). Understanding situation awareness in nursing work: A hybrid concept analysis. Adv Nurs Sci.

[11] Cooper S, Porter J, Peach L (2014). Measuring situation awareness in emergency settings: a systematic review of tools and outcomes. Open Access Emerg Med.

[12] Moher D, Liberati A, Tetzlaff J, Altman DG, Prisma Group. Preferred reporting items for systematic reviews and meta-analyses: the PRISMA statement. PLoS Med. 2009;6(7):e1000097. 10.1371/journal.pmed.1000097 PMC2707599 19621072

[13] Mokkink LB, De Vet HC, Prinsen CA, Patrick DL, Alonso J, Bouter LM (2018). COSMIN risk of bias checklist for systematic reviews of patient-reported outcome measures. Qual Life Res.

[14] Prinsen CA, Mokkink LB, Bouter LM, Alonso J, Patrick DL, De Vet HC (2018). COSMIN guideline for systematic reviews of patient-reported outcome measures. Qual Life Res.

[15] Terwee CB, Prinsen CA, Chiarotto A, Westerman MJ, Patrick DL, Alonso J (2018). COSMIN methodology for evaluating the content validity of patient-reported outcome measures: a Delphi study. Qual Life Res.

[16] Altman DG. Practical statistics for medical research Chapman and Hall. London and New York. 1991.

[17] Gardner AK, Kosemund M, Martinez J. Examining the Feasibility and Predictive Validity of the SAGAT Tool to Assess Situation Awareness Among Medical Trainees. Simulation in healthcare. Journal of the Society for Simulation in Healthcare. 2017;12(1):17-21. 10.1097/sih.0000000000000181. 10.1097/SIH.0000000000000181 27504889

[18] Lavoie P, Cossette S, Pepin J. Testing nursing students' clinical judgment in a patient deterioration simulation scenario: Development of a situation awareness instrument. Nurse Education Today. 2016;38:61-67. 10.1016/j.nedt.2015.12.015. 10.1016/j.nedt.2015.12.015 26749458

[19] Hogan MP, Pace DE, Hapgood J, Boone DC. Use of human patient simulation and the Situation Awareness Global Assessment Technique in practical trauma skills assessment. Journal of Trauma - Injury, Infection and Critical Care. 2006;61(5):1047-1052. 10.1097/01.ta.0000238687.23622.89. 10.1097/01.ta.0000238687.23622.89 17099507

[20] O'Neill TA, White J, Delaloye N, Gilfoyle E. A taxonomy and rating system to measure situation awareness in resuscitation teams. Plos One. 2018;13(5). 10.1371/journal.pone.0196825. 10.1371/journal.pone.0196825 PMC5951547 29758042

[21] Crozier MS, Ting HY, Boone DC, O’Regan NB, Bandrauk N, Furey A, et al. Use of human patient simulation and validation of the team situation awareness global assessment technique (TSAGAT): A multidisciplinary team assessment tool in trauma education. J Surg Educ. 2015;72(1):156–63. 10.1016/j.jsurg.2014.07.009. 10.1016/j.jsurg.2014.07.009 25441262

[22] Jung JJ, Yule S, Boet S, Szasz P, Schulthess P, Grantcharov T. Nontechnical Skill Assessment of the Collective Surgical Team Using the Non-Technical Skills for Surgeons (NOTSS) System. Annals of Surgery. 2020;272(6):1158-1163. 10.1097/SLA.0000000000003250. 10.1097/SLA.0000000000003250 30817354

[23] Yule S, Gupta A, Gazarian D, Geraghty A, Smink DS, Beard J, et al. Construct and criterion validity testing of the Non-Technical Skills for Surgeons (NOTSS) behaviour assessment tool using videos of simulated operations. Br J Surg. 2018;105(6):719–27. 10.1002/bjs.10779. 10.1002/bjs.10779 29601087

[24] Crossley J, Marriott J, Purdie H, Beard JD. Prospective observational study to evaluate NOTSS (Non-Technical Skills for Surgeons) for assessing trainees’ non-technical performance in the operating theatre. Br J Surg. 2011;98(7):1010–20. 10.1002/bjs.7478. 10.1002/bjs.7478 21480195

[25] Yule S, Flin R, Maran N, Rowley D, Youngson G, Paterson-Brown S. Surgeons' non-technical skills in the operating room: reliability testing of the NOTSS behavior rating system. World Journal of Surgery. 2008;32(4):548-556. 10.1007/s00268-007-9320-z. 10.1007/s00268-007-9320-z 18259809

[26] Yule S, Flin R, Paterson-Brown S, Maran N, Rowley D. Development of a rating system for surgeons' non-technical skills. Medical Education. 2006;40(11):1098-1104. 10.1111/j.1365-2929.2006.02610.x. 10.1111/j.1365-2929.2006.02610.x 17054619

[27] Aydın A, Griffin CM, Brunckhorst O, Al-Jabir A, Raison N, Aya H, McIlhenny C, Brewin J, Shabbir M, Palou Redorta J, et al. Non-technical skills for urological surgeons (NoTSUS): development and evaluation of curriculum and assessment scale. World Journal of Urology. 2020. 10.1007/s00345-020-03406-6. 10.1007/s00345-020-03406-6 PMC8217036 32809178

[28] Fletcher G, Flin R, McGeorge P, Glavin R, Maran N, Patey R. Anaesthetists' Non-Technical Skills (ANTS): evaluation of a behavioural marker system. British Journal of Anaesthesia. 2003;90(5):580-588. 10.1093/bja/aeg112. 10.1093/bja/aeg112 12697584

[29] Graham J, Hocking G, Giles E. Anaesthesia Non-Technical Skills: Can anaesthetists be trained to reliably use this behavioural marker system in 1 day? British Journal of Anaesthesia. 2010;104(4):440-445. 10.1093/bja/aeq032. 10.1093/bja/aeq032 20190257

[30] Rutherford J, Flin R, Hellaby M, Caldwell D. Testing the reliability, validity and usability of the prototype anaesthetic non-technical skills-anaesthetic practitioner (ANTS-AP) behaviour rating system. Anaesthesia. 2015;70:32. 10.1111/anae.12962. 10.1111/anae.13127 26152252

[31] van Maarseveen OEC, Ham WHW, Huijsmans RLN, Dolmans RGF, Leenen LPH. Reliability of the assessment of non-technical skills by using video-recorded trauma resuscitations. European journal of trauma and emergency surgery: official publication of the European Trauma Society. 2020. 10.1007/s00068-020-01401-5. 10.1007/s00068-020-01401-5 PMC8825620 32617607

[32] Steinemann S, Berg B, Ditullio A, Skinner A, Terada K, Anzelon K, Ho HC. Assessing teamwork in the trauma bay: Introduction of a modified "nOTECHS" scale for trauma. American Journal of Surgery. 2012;203(1):69-75. 10.1016/j.amjsurg.2011.08.004. 10.1016/j.amjsurg.2011.08.004 22172484

[33] Mishra A, Catchpole K, McCulloch P. The Oxford NOTECHS System: reliability and validity of a tool for measuring teamwork behaviour in the operating theatre. Quality & Safety in Health Care. 2009;18(2):104-108. 10.1136/qshc.2007.024760. 10.1136/qshc.2007.024760 19342523

[34] Robertson ER, Hadi M, Morgan LJ, Pickering SP, Collins G, New S, Griffin D, McCulloch P, Catchpole KC. Oxford NOTECHS II: A modified theatre team non-technical skills scoring system. PloS One. 2014;9(3). 10.1371/journal.pone.0090320. 10.1371/journal.pone.0090320 PMC3942429 24594911

[35] Raison N, Wood T, Brunckhorst O, Abe T, Ross T, Challacombe B, Khan MS, Novara G, Buffi N, Van Der Poel H, et al. Development and validation of a tool for non-technical skills evaluation in robotic surgery—the ICARS system. Surgical Endoscopy. 2017;31(12):5403-5410. 10.1007/s00464-017-5622-x. 10.1007/s00464-017-5622-x 28634630

[36] Kemper PF, van Noord I, de Bruijne M, Knol DL, Wagner C, van Dyck C. Development and reliability of the explicit professional oral communication observation tool to quantify the use of non-technical skills in healthcare. BMJ Qual Saf. 2013;22(7):586–95. 10.1136/bmjqs-2012-001451. 10.1136/bmjqs-2012-001451 PMC3711364 23412933

[37] Loh HP, De Korne DF, Yin SQ, Ang E, Lau Y. Assessment of Scrub Practitioners' List of Intraoperative Non-Technical Skills (SPLINTS) in an Asian Ambulatory Surgical Setting. AORN Journal. 2019;109(4):465-476. 10.1002/aorn.12640. 10.1002/aorn.12640 30919420

[38] Mitchell L, Flin R, Yule S, Mitchell J, Coutts K, Youngson G. Evaluation of the Scrub Practitioners' List of Intraoperative Non-Technical Skills (SPLINTS) system. International Journal of Nursing Studies. 2012;49(2):201-211. 10.1016/j.ijnurstu.2011.08.012. 10.1016/j.ijnurstu.2011.08.012 21974792

[39] Kim J, Neilipovitz D, Cardinal P, Chiu M, Clinch J. A pilot study using high-fidelity simulation to formally evaluate performance in the resuscitation of critically ill patients: The University of Ottawa Critical Care Medicine, High-Fidelity Simulation, and Crisis Resource Management I Study. Critical Care Medicine. 2006;34(8):2167-2174. 10.1097/01.ccm.0000229877.45125.cc. 10.1097/01.CCM.0000229877.45125.CC 16775567

[40] Endsley MR (2021). A Systematic Review and Meta-Analysis of Direct Objective Measures of Situation Awareness: A Comparison of SAGAT and SPAM. Hum Factors.

[41] Schulz C, Krautheim V, Hackemann A, Kreuzer M, Kochs EF, Wagner KJ (2014). Situation awareness errors during critical incidents in anaesthesia and intensive care. Eur J Anaesthesiol.

[42] Vannucci A, Kras JF (2013). Decision making, situation awareness, and communication skills in the operating room. Int Anesthesiol Clin.

[43] Polit DF, Yang FM. Measurement and the measurement of change: a primer for the health professions.: Wolters Kluwer; 2015.

[44] Yoon S, Speyer R, Cordier R, Aunio P, Hakkarainen A. A systematic review evaluating psychometric properties of parent or caregiver report instruments on child maltreatment: part 1: content validity. Trauma Violence Abuse. 2020:1524838019898456. 10.1177/1524838019898456 31928172

[45] Yoon S, Speyer R, Cordier R, Aunio P, Hakkarainen A. A systematic review evaluating psychometric properties of parent or caregiver report instruments on child maltreatment: part 2: internal consistency, reliability, measurement error, structural validity, hypothesis testing, cross-cultural validity, and criterion validity. Trauma Violence Abuse. 2020:1524838020915591. 10.1177/1524838020915591 PMC8739544 32270753

[46] Aminizadeh M, Farrokhi M, Ebadi A, Masoumi GR, Beyrami-Jam M, Khankeh HR (2021). COSMIN Checklist for Systematic Reviews of the Hospital Preparedness Instruments in Biological Events. J Nurs Meas.

[47] Mokkink LB, Terwee CB, Gibbons E, Stratford PW, Alonso J, Patrick DL (2010). Inter-rater agreement and reliability of the COSMIN (COnsensus-based Standards for the selection of health status Measurement Instruments) checklist. BMC Med Res Methodol.

[48] Aydin A, Griffin CM, Brunckhorst O, Al-Jabir A, Raison N, Aya H, et al. Non-technical skills for urological surgeons (NoTSUS): development and evaluation of curriculum and assessment scale. World J Urol. 2020;Epub 10.1007/s00345-020-03406-6 PMC8217036 32809178

[49] DeVellis RF (2003). Scale development: theory and applications.

[50] Rezai M, Kolne K, Bui S, Lindsay S. Measures of workplace inclusion: a systematic review using the COSMIN methodology. J Occup Rehabil. 2020:30(3):420-54. 10.1007/s10926-020-09872-4 31939009

