# Supplementary material for: Measuring situation awareness in health care providers: a systematic review of measurement properties using COSMIN methodology
Source: Syst Rev. 2023 Apr 1;12:60. doi: 10.1186/s13643-023-02220-6 (PMC10067306; doi:10.1186/s13643-023-02220-6)
Supplement: Supplementary file 2 — Additional file 2. Search Strategy. [file 13643_2023_2220_MOESM2_ESM.docx]

**Additional file 2** Search Strategy

| Database | Search Terms (Subject Headings and Free Text Words) | No. of records |
| --- | --- | --- |
| PubMed | (("situation awareness"[tiab] OR "situational awareness"[tiab] OR "clinical competence"[mesh] OR "clinical competence"[tiab] OR "non-technical skills"[tiab] OR "nontechnical skills"[tiab]) AND (psychometrics[mesh] OR psychometrics[tiab] OR reliability[ti] OR validity [ti] OR [validation studies as topic](https://www.ncbi.nlm.nih.gov/mesh/68054928)[mesh]) AND (1966/01/01:2020/12/31[dp])) | 2,171 |
| Web of science | (TS="situation awareness" OR TS="situational awareness" OR TS="nontechnical skills" OR TS="non-technical skills") AND (TS=psychometrics OR TI= psychometrics OR TS= validity OR TS=reliability OR TS="[validation studies as topic](https://www.ncbi.nlm.nih.gov/mesh/68054928)") AND PY=(1975-2020) | 1,014 |
| Scopus | (TITLE-ABS-KEY("situation awareness") OR TITLE-ABS-KEY("situational awareness") OR TITLE-ABS-KEY("non-technical skills") OR TITLE-ABS-KEY("nontechnical skills")) AND (INDEXTERMS (psychometrics) OR TITLE-ABS-KEY (psychometrics) OR INDEXTERMS (validity) OR TITLE-ABS-KEY (validity) OR INDEXTERMS (reliability) OR TITLE-ABS-KEY (reliability) OR INDEXTERMS (validation studies as topic)) AND (PUBYEAR > 1972 AND PUBYEAR < 2021) | 1,123 |
| Embase | ('situation awareness*'/de OR 'situational awareness*':ti,ab,kw OR 'nontechnical skill*':ti,ab,kw OR 'non-technical skill*':ti,ab,kw) AND ('psychometr*'/de OR psychometric*:ti,ab,kw OR valid*:ti,ab,kw OR 'validation studies as topic'/de OR reliab*:ti,ab,kw) AND ([1-1-1947]/sd NOT [1-1-2021]/sd) | 763 |
