# Supplementary material for: Measuring situation awareness in health care providers: a systematic review of measurement properties using COSMIN methodology
Source: Syst Rev. 2023 Apr 1;12:60. doi: 10.1186/s13643-023-02220-6 (PMC10067306; doi:10.1186/s13643-023-02220-6)
Supplement: Supplementary file 3 — Additional file 3. Guidance of rating the results of single studies based on COSMIN methodology. [file 13643_2023_2220_MOESM3_ESM.docx]

**Additional file 3** Guidance of rating the results of single studies based on COSMIN methodology

| Additional file 3-1 Guidance for giving a sufficient (+) rating for the 10 criteria for good content validity of instrument | | | | |
| --- | --- | --- | --- | --- |
|  | PROM development study | Content validity study | Reviewers’ rating | Remarks |
| 1 | The construct of interest is clearly described (i.e. ‘very good’ rating of box 1 standard 1), the origin of construct is clear (i.e. ‘very good’ rating of box 1 standard 2) and there is evidence from concept elicitation, literature, or professionals that at least 85% of the items refer to the construct of interest. | Professionals rated the relevance of the items for the construct of interest in a content validity study that was not inadequate (i.e. ‘very good’, ‘adequate’ or ‘doubtful’ rating for quality of relevance study in box 2d, standards 22‐26) and found at least 85% of the items relevant for the construct. | Reviewers consider at least 85% of the items relevant for the construct of interest. | Every PROM item should measure a defined facet of the construct of interest, within the conceptual framework. PROM items should also be specific for the construct of interest, i.e. they should not measure a co‐ existing, but separate construct. For example, an item in a fatigue questionnaire such as “my muscles are weak” is relevant to fatigue but not specific for fatigue. Someone with MS may answer this question in the affirmative but not experiencing fatigue. There should also be no unnecessary items (too many items, except for a large scale item bank that will be used for computer adaptive testing). When a total PROM score is evaluated, each subscale (domain) should be relevant for the construct that the total PROM intends to measure. Professionals can best ensure that items are consistent with the theory, conceptual framework or disease model that was used to define the construct of interest. |
| 2 | The target population of interest is | Patients rated the relevance of the | Reviewers consider at least 85% of | The relevance of the items for the |
|  | clearly described (i.e. ‘very good’ rating box 1 standard 3) and representative patients were involved in the elicitation of relevant items (i.e. ‘very good’ or ‘adequate’ rating box 1 standard 5) and concept elicitation (‘worst score counts’ box 1a standards 6‐ 13) was not inadequate. If it is doubtful whether the study was performed in a sample representing the target population, we recommend to give an indeterminate (?) rating. | items for them in a content validity study that was not inadequate (rating for quality of relevance study, box 2a standards 1‐7) and found at least 85% of the items relevant for them. | the items relevant for the population of interest. | target population can best be judged by patients. Some items may be relevant to only a small number of patients but they are necessary to capture the full range of patient experiences. |
| 3 | The context of use of interest is clearly described (i.e. ‘very good’ rating box 1 standard 4). | Professionals rated the relevance of the items for the context of use of interest in a content validity study that was not inadequate (rating for quality of relevance study, i.e. box 2d, standards 22‐26) and found at least 85% of the items relevant for the context of use. | Reviewers consider at least 85% of the items relevant for the context of use of interest. | It should especially be clear whether the PROM is suitable for use in research and/or clinical practice. Professionals are considered to be more knowledgeable about the context of use of the PROM than patients. |
| 4 | A justification is provided for the response options. | Patients or professionals rated the appropriateness of the response options in a content validity study that was not inadequate (rating for quality of relevance study, i.e. box 2a standards 1‐7 or box 2d standards 22‐26) and found at least 85% of the response options | Reviewers consider the at least 85% of the response options appropriate for the construct, population, and context of use of interest. | The response options should be appropriate for the construct, population, and context of use of interest. For example, if the construct is pain intensity, the response options should measure intensity, not frequency. Also, a reasonable range of responses |
|  |  | relevant. |  | should be provided for measuring the construct of interest. |
| 5 | A justification is provided for the recall period. | Patients or professionals rated the appropriateness of the recall period in a content validity study that was not inadequate (rating for quality of relevance study, i.e. box 2a standards 1‐7 or box 2d standards 22‐26) and found the recall period appropriate. | Reviewers consider the recall period appropriate for the construct, population, and context of use of interest. | The recall period can be important for measuring the construct, for example, whether there is no recall period (do you feel depressed now?) or whether the recall period is 1 week (did you feel depressed last week?). Different recall periods may be important, depending on the context. However, sometimes is does not matter whether the recall period is e.g. 1 or 2 weeks. |
| 6 | Patients were asked about the comprehensiveness of the PROM in the concept elicitation phase or in a cognitive interview study that was not inadequate (rating for quality of comprehensiveness study, i.e. box 1a standards 6‐13, or box 1b standards 26‐35) and no key concepts were missing. | Patients or professionals were asked about the comprehensiveness of the PROM in a content validity study that was not inadequate (rating for quality of comprehensiveness study, i.e. box 2b standards 8‐14, or box 2e standards 27‐31) and no key concepts were missing. | Reviewers consider the PROM comprehensive for the construct, population and context of use of interest. | The items should cover the full breadth of the construct of interest. However, there are often good reasons for not including all content suggested by patients in a PROM, for example because an item (or domain) is considered to be outside the scope of the PROM. When a total PROM score is evaluated, the subscales (domain) together should cover the full breath of the construct that the total PROM intends to measure. |
| 7 | Patients were asked about the comprehensibility of the instructions (including recall period) in a cognitive interview study that was not inadequate | Patients were asked about the comprehensibility of the instructions (including recall period) in a content validity study that was not inadequate (rating for |  |  |
|  | (rating for quality of comprehensibility study, i.e. box 1b standards 16‐25) and problems were adequately addressed. | quality of comprehensibility study, box 2c standards 15‐21) and no important problems were found. |  |  |
| 8 | Patients were asked about the comprehensibility of the items and response options (including wording of the items and response options) in a cognitive interview study that was not inadequate (rating for quality of comprehensibility study, box 1b standards 16‐25) and problems were adequately addressed. | Patients were asked about the comprehensibility of the items and response options in a content validity study that was not inadequate (rating for quality of comprehensibility study, box 2c standards 15‐21) and no important problems were found for at least 85% of the items and response options. |  |  |
| 9 |  |  | Reviewers consider at least 85% of the items and response options appropriately worded. | Consider aspects such as reading level (a scale should not require reading skills beyond that of a 12‐ year old), ambiguous items, double‐barrelled questions, jargon, value‐laden words, and length or items [97]. |
| 10 |  |  | Reviewers consider at least 85% of the response options matching the questions. | The response options should be appropriate to the question asked and should be linguistically linked to the item content. |

| Additional file 3-2 Guidance for determining the RELEVANCE RATING, COMPREHENSIVENESS RATING, and COMPREHENSIBILITY RATING per study | | | | |
| --- | --- | --- | --- | --- |
|  | RELEVANCE | COMPREHENSIVENESS | COMPREHENSIBILITY | |
|  |  |  | PROM development study AND content validity studies | Reviewers rating |
| + | At least criteria 1 and 2 are rated + AND at least two of the other three criteria on relevance are rated +  Criteria 1 and 2 (relevance for construct and population) are considered the most important criteria and therefore they need to be rated +. A maximum of 1 criterion rated – is allowed, but reviewers can also rate ± in that case. | Rating of criterion 6 | At least criterion 8 is rated + and criterion 7 is NOT rated ‐ Criterion 8 is considered the most important, but for a sufficient rating criterion 7 should NOT be rated ‐ (it may be rated ?). | Both criteria 9 and 10 are rated + |
| ‐ | at least criteria 1 and 2 are rated ‐ AND at least two of the other three criteria on relevance are rated ‐ | Rating of criterion 6 | Criterion 8 is rated – (independent of the rating for criterion 7) | Both criteria 9 and 10 are rated ‐ |
| ? | At least two of the criteria are rated ? | Rating of criterion 6 | Criterion 8 is rated ? (independent of the rating for criterion 7) | At least one of the criteria is rated ? |
| ± | All other situations | Rating of criterion 6 | Criterion 8 is rated + and criterion 7 is rated ‐ | One criterion is rated + and one is rated ‐ |

| **Additional file 3-3** Updated criteria for good measurement properties | | |
| --- | --- | --- |
| Measurement Properties | Rating | Criteria |
| Structural validity | + | **CTT** CFA: CFI or TLI or comparable measure > 0.95 OR RMSEA < 0.06 OR SRMR < 0.08^a^ |
|  |  | **IRT/Rasch** No violation of unidimensionality^b^: CFI or TLI or comparable measure > 0.95 OR RMSEA < 0.06 OR SRMR < 0.08 *AND* no violation of local independence: residual correlations among the items after controlling for the dominant factor < 0.20 OR Q3’s < 0.37 *AND* no violation of monotonicity: adequate looking graphs OR item scalability > 0.30 *AND* adequate model fit IRT: χ^2^ > 0.001 Rasch: infit and outfit mean squares ≥ 0.5 and ≤ 1.5 OR Z-standardized values > −2 and < 2 |
|  | ? | CTT: not all information for ‘+’ reported IRT/Rasch: model fit not reported |
|  | − | Criteria for ‘+’ not met |
| Internal consistency | + | At least low evidence^c^ for sufficient structural validity^d^ AND Cronbach’s alpha(s) ≥ 0.70 for each unidimensional scale or subscale^e^ |
|  | ? | Criteria for “At least low evidence^c^ for sufficient structural validity^d^” not met |
|  | − | At least low evidence^c^ for sufficient structural validity^d^ AND Cronbach’s alpha(s) < 0.70 for each unidimensional scale or subscale^e^ |
| Reliability | + | ICC or weighted Kappa ≥ 0.70 |
|  | ? | ICC or weighted Kappa not reported |
|  | − | ICC or weighted Kappa < 0.70 |
| Measurement error | + | SDC or LoA < MIC^d^ |
|  | ? | MIC not defined |
|  | − | SDC or LoA > MIC^d^ |
| Hypotheses testing for construct validity | + | The result is in accordance with the hypothesis^f^ |
|  | ? | No hypothesis defined (by the review team) |
|  | − | The result is not in accordance with the hypothesis^f^ |
| Cross-cultural validity\measurement invariance | + | No important differences found between group factors (such as age, gender, language) in multiple group factor analysis OR no important DIF for group factors (McFadden’s R^2^ < 0.02) |
|  | ? | No multiple group factor analysis OR DIF analysis performed |
|  | − | Important differences between group factors OR DIF was found |
| Criterion validity | + | Correlation with gold standard ≥ 0.70 OR AUC ≥ 0.70 |
|  | ? | Not all information for ‘+’ reported |
|  | − | Correlation with gold standard < 0.70 OR AUC < 0.70 |
| Responsiveness | + | The result is in accordance with the hypothesis^f^ OR AUC ≥ 0.70 |
|  | ? | No hypothesis defined (by the review team) |
|  | − | The result is not in accordance with the hypothesis^f^ OR AUC < 0.70 |

1. The criteria are based on, e.g., Terwee et al. and Prinsen et al.
2. AUC area under the curve, CFA confirmatory factor analysis, CFI comparative fit index, CTT classical test theory, DIF differential item functioning, ICC intraclass correlation coefficient, IRT item response theory, LoA limits of agreement, MIC minimal important change, RMSEA root mean square error of approximation, SEM standard error of measurement, SDC smallest detectable change, SRMR standardized root mean residuals, TLI Tucker–Lewis index
3. “+” = sufficient, “−” = insufficient, “?” = indeterminate
4. aTo rate the quality of the summary score, the factor structures should be equal across studies
5. ^b^Unidimensionality refers to a factor analysis per subscale, while structural validity refers to a factor analysis of a (multidimensional) patient-reported outcome measure
6. ^c^As defined by grading the evidence according to the GRADE approach
7. ^d^This evidence may come from different studies
8. ^e^The criteria ‘Cronbach alpha < 0.95’ was deleted, as this is relevant in the development phase of a PROM and not when evaluating an existing PROM
9. ^f^The results of all studies should be taken together and it should then be decided if 75% of the results are in accordance with the hypotheses
