# Supplementary material for: Measuring situation awareness in health care providers: a systematic review of measurement properties using COSMIN methodology
Source: Syst Rev. 2023 Apr 1;12:60. doi: 10.1186/s13643-023-02220-6 (PMC10067306; doi:10.1186/s13643-023-02220-6)
Supplement: Supplementary file 4 — Additional file 4. Guidance of grading the evidence quality for each measurement property based on COSMIN methodology. [file 13643_2023_2220_MOESM4_ESM.docx]

**Additional file 4** Guidance of grading the evidence quality for each measurement property based on COSMIN methodology

**Additional file 4-1** Grading the quality of evidence on content validity (modified GRADE approach)

| Study design | Quality of evidence | Lower if |
| --- | --- | --- |
| At least 1 content validity study | High | Risk of bias ‐1 Serious ‐2 Very serious ‐3 Extremely serious Inconsistency ‐1 Serious ‐2 Very serious Indirectness ‐1 Serious ‐2 Very serious |
| No content validity studies | Moderate |  |
|  | Low |  |
|  | Very low |  |

**Additional file 4-2** Modified GRADE approach for grading the quality of evidence other measurement properties

| Quality of evidence | Lower if |
| --- | --- |
| High | Risk of bias  −1 Serious  −2 Very serious  −3 Extremely serious Inconsistency  −1 Serious  −2 Very serious Imprecision  −1 total *n* = 50–100  −2 total *n* < 50 Indirectness  −1 Serious  −2 Very serious |
| Moderate |  |
| Low |  |
| Very low |  |

The starting point is the assumption that the evidence is of high quality. The quality of evidence is subsequently downgraded with one or two levels for each factor (i.e., risk of bias, inconsistency, imprecision, indirectness) to moderate, low, or very low when there is risk of bias (low study quality), (unexplained) inconsistency in results, or indirect results. Information on how to downgrade is described in detail in the COSMIN user manual. *n* = sample size

**Additional file 4-3** Definitions of quality levels

| Quality level | Definition |
| --- | --- |
| High | We are very confident that the true measurement property lies close to that of the estimate of the measurement property |
| Moderate | We are moderately confident in the measurement property estimate: the true measurement property is likely to be close to the estimate of the measurement property, but there is a possibility that it is substantially different |
| Low | Our confidence in the measurement property estimate is limited: the true measurement property may be substantially different from the estimate of the measurement property |
| Very low We | have very little confidence in the measurement property estimate: the true measurement property is likely to be substantially different from the estimate of the measurement property |

These definitions were adapted from the GRADE approach. Information on how to downgrade is described in detail in the COSMIN user manual. [Additional file 4-1; Additional file 4-2]
