# Supplementary material for: Measuring situation awareness in health care providers: a systematic review of measurement properties using COSMIN methodology
Source: Syst Rev. 2023 Apr 1;12:60. doi: 10.1186/s13643-023-02220-6 (PMC10067306; doi:10.1186/s13643-023-02220-6)
Supplement: Supplementary file 5 — Additional file 5. Methodological quality assessment 0f studies on psychometric properties of the included instruments. [file 13643_2023_2220_MOESM5_ESM.docx]

| **Additional file 5** Methodological quality assessment 0f studies on psychometric properties of the included instruments | | | | | | | | | | | | | | | | | | |
| --- | --- | --- | --- | --- | --- | --- | --- | --- | --- | --- | --- | --- | --- | --- | --- | --- | --- | --- |
| Instrument | Reference | Development Study Quality | | | Content Validity | | | | | Structural validity | Internal consistency | Cross-cultural validity | Reliability | Measurement error | Criterion validity | Hypotheses testing for construct validity | | Responsiveness |
|  |  | Item generation | Cognitive Interview | | Asking patients | | | Asking experts | |  |  |  |  |  |  | Convergent validity | Known-groups validity |  |
|  |  | Relevance | Compre-hensibility | Compre-hensiveness | Relevance | Compre-hensiveness | Compre-hensibility | Relevance | Compre-hensiveness |  |  |  |  |  |  |  |  |  |
| SAGAT | Dishman  2020 | Adequate | Inadequate | Doubtful | Adequate | Inadequate | Inadequate | Adequate | Inadequate | NR | NR | NR | NR | NR | NR | NR | | NR |
|  | Gardner  2017 | Doubtful | Inadequate | Inadequate | NR | NR | NR | NR | NR | NR | NR | NR | NR | NR | Doubtful | NR | | NR |
|  | Lavoie  2016 | Very good | Doubtful | Doubtful | NR | NR | NR | Very good | Very good | NR | Inadequate | NR | NR | NR | NR | NR | | NR |
|  | Hogan  2006 | Inadequate | Inadequate | Inadequate | NR | NR | NR | Inadequate | Inadequate | NR | Inadequate | NR | NR | NR | NR | Inadequate |  | NR |
| Unnamed^a^ | O'Neill  2018 | Very good | Doubtful | Doubtful | NR | NR | NR | Adequate | Adequate | NR | NR | NR | NR | NR | inadequate | NR | NR | NR |
| TSAGAT | Crozier  2015 | Adequate | Doubtful | Doubtful | NR | NR | NR | NR | NR | NR | NR | NR |  | NR | NR | Inadequate | Doubtful | NR |
| Unnamed^b^ | Frere  2017 | Doubtful | Inadequate | Inadequate | NR | NR | NR | NR | NR | NR | Very good | NR | NR | NR | NR | NR | NR | NR |
| NOTSS | Jung  2020 | NR | NR | NR | NR | NR | NR | NR | NR | NR | NR | NR | NR | NR | NR | NR | Doubtful | NR |
|  | Yule  2018 | NR | NR | NR | NR | NR | NR | NR | NR | Very good | Very good | NR | NR | NR | inadequate | NR | NR | NR |
|  | Crossley  2011 | NR | NR | NR | NR | NR | NR | Adequate | Adequate | Very good | Very good | NR | NR | NR | NR | NR | NR | NR |
|  | Yule  2008 | NR | NR | NR | NR | NR | NR | NR | NR | NR | Very good | NR | NR | NR | NR | NR | | NR |
|  | Yule  2006 | Very good | Adequate | Adequate | NR | NR | NR | NR | NR | NR | NR | NR | NR | NR | NR | NR | | NR |
| NoTSUS | Aydın  2020 | Adequate | Inadequate | Inadequate | NR | NR | NR | NR | NR | NR | NR | NR | NR | NR | Very good | NR | | NR |
| ANTS | Fletcher  2003 | Very good | Adequate | Adequate | NR | NR | NR | Inadequate | Adequate | NR | Doubtful | NR | NR | NR | NR | NR | | NR |
|  | Graham  2010 | NR | NR | NR | NR | NR | NR | NR | NR | NR | Very good | NR | NR | NR | NR | NR | | NR |
| ANTS-AP | Rutherford  2015 | Very good | Adequate | Adequate | NR | NR | NR | Inadequate | Adequate | NR | Very good | NR | Adequate | NR | NR | NR | | NR |
| T-NOTECHS | van Maarseveen  2020 | NR | NR | NR | NR | NR | NR | NR | NR | NR | NR | NR | Doubtful | NR | NR | NR | | NR |
|  | Steinemann 2012 | Adequate | Inadequate | Doubtful | NR | NR | NR | NR | NR | NR | NR | NR | NR | NR | NR | NR | | NR |
| NOTECHS | Mishra  2009 | Adequate | Doubtful | Doubtful | NR | NR | NR | Doubtful | Doubtful | NR | NR | NR | Inadequate | NR | NR | Inadequate | NR | NR |
| NOTECHS II | Robertson  2014 | Adequate | Doubtful | Doubtful | NR | NR | NR | Doubtful | Doubtful | NR | NR | NR | NR | NR | NR | NR | Adequate | NR |
| ICARS | Raison  2017 | Adequate | Inadequate | Doubtful | NR | NR | NR | Doubtful | Doubtful | NR | Very good | NR | NR | NR | NR | NR | Very good | NR |
| EPOC | Kemper  2013 | Adequate | Inadequate | Doubtful | NR | NR | NR | NR | NR | NR | NR | NR | NR | Adequate | NR | NR | | NR |
| SPLINTS | Loh  2019 | NR | NR | NR | NR | NR | NR | Very good | Very good | NR | Very good | NR | Doubtful | NR | NR | Inadequate | NR | NR |
|  | Mitchell  2011 | Very good | Doubtful | Doubtful | NR | NR | NR | NR | NR | NR | NR | NR | NR | NR | NR | NR | NR | NR |
| Ottawa GRS | Kim  2006 | Doubtful | Inadequate | Doubtful | NR | NR | NR | Inadequate | Inadequate | NR | Inadequate | NR | NR | NR | NR | NR | | NR |

Notes.

The full name of instruments: Situation Awareness Global Assessment Technique (SAGAT); Team Situation Awareness Global Assessment Technique (TSAGAT); Non-Technical Skills for Surgeons tool (NOTSS); Non-technical Skills for Urological Surgeons (NoTSUS); Anaesthetists' Non-Technical Skills system (ANTS); Anaesthetic Non-Technical Skills for Anaesthetic Practitioners system (ANTS-AP); Trauma Non-Technical Skills (T-NOTECHS) Tool; Oxford Non-Technical Skills scale (NOTECHS); Oxford Non-Technical Skills scale (NOTECHS II); Interpersonal and Cognitive Assessment for Robotic Surgery rating system (ICARS); Explicit professional oral communication tool (EPOC); Scrub Practitioners’ List of Intraoperative Non-Technical Skills (SPLINTS); Ottawa Global Rating Scale (GRS)

^a^Team resuscitation situation awareness tool

^b^Situation awareness (SA) assessment tool

NR= not reported
