# Supplementary material for: Measuring situation awareness in health care providers: a systematic review of measurement properties using COSMIN methodology
Source: Syst Rev. 2023 Apr 1;12:60. doi: 10.1186/s13643-023-02220-6 (PMC10067306; doi:10.1186/s13643-023-02220-6)
Supplement: Supplementary file 6 — Additional file 6. Quality of content validity (per PROM development and Content validity study, and Rating of reviewers) and other psychometric properties per study. [file 13643_2023_2220_MOESM6_ESM.docx]

| **Additional file 6** Quality of content validity (per PROM development and Content validity study, and Rating of reviewers) and other psychometric properties per study | | | | | | | | | | | |
| --- | --- | --- | --- | --- | --- | --- | --- | --- | --- | --- | --- |
| Instrument | Reference | Relevance | | | Comprehensiveness | | | Comprehensibility | | | other psychometric properties |
|  |  | PROM development study | Content validity study | Rating of reviewers | PROM development study | Content validity study | Rating of reviewers | PROM development study | Content validity study | Rating of reviewers |  |
| SAGAT | Dishman  2020 | + | + | + | ? | ? | + | ? | ? | + | NR |
|  | Gardner  2017 | + | ± | + | - | - | - | - | - | ± | Criterion validity (+) |
|  | Lavoie  2016 | + | ± | + | + | ? | + | ? | - | + | Internal consistency (-) |
|  | Hogan  2006 | ? | ? | ? | - | - | + | ? | - | ? | Internal consistency (?)  Convergent validity (?) |
| Unnamed^a^ | O'Neill  2018 | + | ± | + | + | + | + | + | ? | + | Criterion validity (?)  Inter-rater reliability |
| TSAGAT | Crozier  2015 | ? | ? | ± | + | ? | + | + | ? | + | Convergent validity (+)  Known-groups validity (+)  Inter-rater reliability |
| Unnamed^b^ | Frere  2017 | ? | ? | ? | ? | ? | ? | ? | ? | ? | Internal consistency (+)  Inter-rater reliability |
| NOTSS | Jung  2020 | NR | NR | NR | NR | NR | NR | NR | NR | NR | Known-groups validity (+)  Inter-rater reliability |
|  | Yule  2018 | NR | NR | NR | NR | NR | NR | NR | NR | NR | Structural validity (-)  Internal consistency (+)  Criterion validity (?) |
|  | Crossley  2011 | NR | NR | NR | + | ? | ? | NR | NR | NR | Structural validity (?)  Internal consistency (?) |
|  | Yule  2008 | NR | NR | NR | NR | NR | NR | NR | NR | NR | Internal consistency (?)  Inter-rater reliability |
|  | Yule  2006 | + | ? | + | - | ? | + | ? | ? | + | NR |
| NoTSUS | Aydın  2020 | ? | ? | ? | ? | ? | ? | ? | ? | ? | Criterion validity (+)  Inter-rater reliability |
| ANTS | Fletcher  2003 | + | ? | + | + | ? | + | + | ? | + | Internal consistency (+)  Inter-rater reliability |
|  | Graham  2010 | NR | NR | NR | NR | NR | NR | NR | NR | NR | Internal consistency (+)  Inter-rater reliability |
| ANTS-AP | Rutherford  2015 | + | ? | ? | + | + | + | + | ? | + | Internal consistency (+)  Reliability (-)  Inter-rater reliability |
|  | van Maarseveen  2020 | NR | NR | NR | NR | NR | NR | NR | NR | NR | Reliability (+)  Inter-rater reliability |
|  | Steinemann  2012 | + | ? | + | ? | ? | - | - | ? | + | Inter-rater reliability |
| NOTECHS | Mishra  2009 | + | ? | + | + | ? | + | ? | ? | + | Reliability (?)  Convergent validity (+)  Inter-rater reliability |
| NOTECHS II | Robertson  2014 | + | ? | + | + | ? | + | ? | ? | + | Known-groups validity (+)  Inter-rater reliability |
| ICARS | Raison  2017 | + | ? | + | ? | ? | + | - | ? | + | Internal consistency (+)  Inter-rater reliability |
| EPOC | Kemper  2013 | + | ? | + | ? | ? | ? | ? | ? | ? | Measurement error (?)  Inter-rater reliability |
| SPLINTS | Loh  2019 | NR | NR | NR | + | + | ? | NR | NR | NR | Internal consistency (+)  Reliability (+)  Convergent validity (+)  Inter-rater reliability |
|  | Mitchell  2011 | + | ? | + | + | ? | + | + | ? | - | NR |
| Ottawa GRS | Kim  2006 | ? | ? | ? | - | ? | - | ? | ? | ± | Internal consistency (?)  Inter-rater reliability |

Notes.

The full name of instruments: Situation Awareness Global Assessment Technique (SAGAT); Team Situation Awareness Global Assessment Technique (TSAGAT); Non-Technical Skills for Surgeons tool (NOTSS); Non-technical Skills for Urological Surgeons (NoTSUS); Anaesthetists' Non-Technical Skills system (ANTS); Anaesthetic Non-Technical Skills for Anaesthetic Practitioners system (ANTS-AP); Trauma Non-Technical Skills (T-NOTECHS) Tool; Oxford Non-Technical Skills scale (NOTECHS); Oxford Non-Technical Skills scale (NOTECHS II); Interpersonal and Cognitive Assessment for Robotic Surgery rating system (ICARS); Explicit professional oral communication tool (EPOC); Scrub Practitioners’ List of Intraoperative Non-Technical Skills (SPLINTS); Ottawa Global Rating Scale (GRS)

^a^Team resuscitation situation awareness tool

^b^Situation awareness (SA) assessment tool

Rating scale: Sufficient (+), Insufficient (–), Inconsistent (±), Indeterminate (?) [see Additional file 3]

NR= not reported
